# Masupirdine (SUVN‐502): Phase‐3 Study for the Potential Treatment of Agitation in Patients with Dementia of the Alzheimer’s Type, an Update

**DOI:** 10.1002/alz.087894

**Published:** 2025-01-09

**Authors:** Ramakrishna Nirogi, Renny Abraham, Jyothsna Ravula, Satish Jetta, Vijay Benade, Anil Shinde, Mohammed Abdul Rasheed, Rajesh Kumar Badange, Vinod Kumar Goyal, Veera Raghava Chowdary Palacharla, Ramkumar Subramanian, Pradeep Jayarajan

**Affiliations:** ^1^ Suven Life Sciences, Hyderabad, Telangana India

## Abstract

**Background:**

Alzheimer’s disease (AD) agitation is a distressing neuropsychiatric symptom characterized by excessive motor activity, verbal aggression, or physical aggression. Agitation is one of the causes of caregiver distress, increased morbidity and mortality, and early institutionalization in patients with AD. Current medications used for the management of agitation have modest efficacy and have substantial side effects. Therefore, there remains an urgent clinical priority for effective and safe treatments for agitation in AD.

Serotonin‐6 (5‐HT_6_) receptors are widely expressed in the brain regions including the cortex, dorsal hippocampus, and striatum; brain areas centrally involved in mood and behavior. Blockade of 5‐HT_6_ receptors may have a potential therapeutic utility in managing agitation. Masupirdine is a potent and selective 5‐HT_6_ receptor antagonist and is being developed as a potential treatment for agitation associated with AD.

**Method:**

Masupirdine was evaluated in animal models of aggressive behaviors like resident‐intruder task and dominant‐submissive assay. The cognitive and motor performances were assessed using the alternating lever cyclic ration schedule.

Subgroup analyses of a Phase‐2, 26‐week proof‐of‐concept clinical study (NCT02580305) based on the neuropsychiatric inventory scale were carried out.

A Phase‐3, double‐blind, randomized, placebo‐controlled, global study is in progress to explore the efficacy, safety, tolerability, and pharmacokinetics of masupirdine in patients with agitation in dementia of the Alzheimer’s type (NCT05397639 and EudraCT 2021‐003405‐22).

**Result:**

Oral administration of masupirdine significantly (p<0.05) attenuated aggressive behaviors in the resident‐intruder task and dominant‐submissive assay. In the alternating lever cyclic ration schedule, no changes were observed in the cognitive and motor performances after treatment with Masupirdine (p>0.05).

Potential beneficial effects in reducing agitation/aggression symptoms were observed in the post hoc analyses of the Phase‐2 study of masupirdine in AD patients.

Updates from the ongoing Phase‐3 study (NCT05397639 and EudraCT 2021‐003405‐22) will be presented in the poster.

**Conclusion:**

Outcomes from animal models of aggression and subgroup analysis of the Phase‐2 study suggest that masupirdine may alleviate agitation. The Phase‐3 study results may inform the therapeutic utility of masupirdine for the treatment of Alzheimer’s agitation. Phase‐3 study data readout is anticipated in Q1/Q2 2025.

